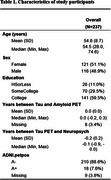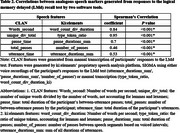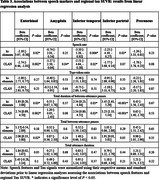# Automated Speech Features from Logical Memory Delayed Recall Test Relate to Early Tau PET Burden

**DOI:** 10.1002/alz70856_102373

**Published:** 2025-12-24

**Authors:** Zexu Li, Christina B. Young, Cody Karjadi, Alexandra König, Louisa Schwed, Nicklas Linz, Johannes Tröger, Julia Peterson, Jinying Chen, Ting Fang Alvin Ang, Rhoda Au

**Affiliations:** ^1^ Dept of Anatomy & Neurobiology, Boston University Chobanian & Avedisian School of Medicine, Boston, MA, USA; ^2^ Department of Neurology and Neurological Sciences, Stanford University School of Medicine, Stanford, CA, USA; ^3^ Framingham Heart Study, Framingham, MA, USA; ^4^ ki:elements GmbH, Saarbrücken, Germany; ^5^ Department of Medicine/Section of Preventive Medicine and Epidemiology, Boston University Chobanian & Avedisian School of Medicine, Boston, MA, USA; ^6^ Data Science Core, Boston University Chobanian & Avedisian School of Medicine, Boston, MA, USA; ^7^ Slone Epidemiology Center, Boston University Chobanian & Avedisian School of Medicine, Boston, MA, USA; ^8^ Department of Anatomy & Neurobiology, Boston University Chobanian & Avedisian School of Medicine, Boston, MA, USA; ^9^ Biomedical Genetics, Department of Medicine, Boston University Medical School, Boston, MA, USA; ^10^ Department of Epidemiology, Boston University School of Public Health, Boston, MA, USA; ^11^ Department of Neurology, Boston University Chobanian & Avedisian School of Medicine, Boston, MA, USA; ^12^ Framingham Heart Study, Boston University Chobanian & Avedisian School of Medicine, Boston, MA, USA

## Abstract

**Background:**

Speech features (e.g., between‐utterance pause duration, speech rate) derived from Logical Memory Delay Recall (LMd) tests were shown to be associated with early tau burden. Various software tools can generate these features but may use different methods. We aim to validate the robustness of speech features by comparing analogous features generated by two software tools and assessing their associations with tau pathology.

**Method:**

We analyzed data from Framingham Heart Study participants who completed amyloid and tau PET imaging within a year of their LMd test. Five speech features were generated using ki:elements’ SIGMA platform and the CLAN software. Ki:elements’ tool derived three features (duration of utterances, duration of between‐utterance pauses, number of between‐utterance pauses) fully automatically from voice recordings. Other Ki:elements features and all CLAN features were derived from manual transcription. Correlations between ki:elements and CLAN features were assessed by Spearman's correlation coefficients. Associations between speech features and tau standardized uptake value ratio (SUVR) in five brain regions were examined using multiple linear regression, adjusting for age, sex, education, and amyloid status.

**Result:**

Data from 237 participants (mean age: 54.6±8.7 years; 51.1% female) were analyzed (Table 1). All pairs of speech features were correlated (*p* <0.001, Table 2). They also demonstrated compatible patterns in their associations with tau SUVR, showing consistency in the direction of association across all cases and in significance levels in most cases (Table 3). In particular, longer between‐utterance pause duration was associated with higher tau SUVR in entorhinal (Ki:elements: beta=1.93, *p* = 0.03;CLAN: beta=2.74, *p* = 0.002), inferior temporal (IT) (Ki:elements: beta=2.34, *p* = 0.03; CLAN: beta=2.34, *p* = 0.03), and inferior parietal (IP) (Ki:elements: beta=3.45, *p* <0.001; CLAN: beta=3.23, *p* <0.001) regions. More words per second was associated with reduced tau SUVR in entorhinal (Ki:elements: beta=‐2.18, *p* = 0.02; CLAN: beta=‐2.74, *p* = 0.002) and IT (Ki:elements: beta=‐3.10, *p* = 0.006; CLAN: beta=‐2.51, *p* = 0.02) regions.

**Conclusion:**

Simple speech features (including fully automated ones) generated using two methods exhibited compatible patterns in their associations with early tau burden. Other automated linguistic and semantic features will be further investigated. With continued validation, automated speech features have the potential to offer a scalable approach with limited loss of findings relative to manual transcriptions.